# The discovery of group 2 innate lymphoid cells has changed the concept of type 2 immune diseases

**DOI:** 10.1093/intimm/dxab063

**Published:** 2021-09-09

**Authors:** Tetsuro Kobayashi, Yasutaka Motomura, Kazuyo Moro

**Affiliations:** 1 Laboratory for Innate Immune Systems, RIKEN Center for Integrative Medical Sciences (IMS), 1-7-22 Suehiro-cho, Tsurumi-ku, Yokohama, Kanagawa 230-0045, Japan; 2 Laboratory for Innate Immune Systems, Department of Microbiology and Immunology, Graduate School of Medicine, Osaka University, 2-2 Yamadaoka, Suita-shi, Osaka 565-0871, Japan; 3 Laboratory for Innate Immune Systems, Immunology Frontier Research Center (iFReC), Osaka University, 3-1, Yamadaoka, Suita-shi, Osaka 565-0871, Japan; 4 Laboratory for Innate Immune Systems, Graduate School of Frontier Biosciences, Osaka University, 1-3 Yamadaoka, Suita-shi, Osaka 565-0871, Japan; 5 Integrated Frontier Research for Medical Science Division, Institute for Open and Transdisciplinary Research Initiatives (OTRI), Osaka University, 2-2 Yamadaoka, Suita-shi, Osaka 565-0871, Japan

**Keywords:** allergy, group 2 innate lymphoid cell, *Taishitsu*

## Abstract

Group 2 innate lymphoid cells (ILC2s), discovered in 2010, have been recognized as immune cells with unique functions and their involvement in various diseases has been clarified. Before 2010, the antigen-specific response was a primary focus of immunology research, and immune responses were considered almost equivalent to biological responses to foreign antigens. However, with the emergence of ILC2s, the importance of ‘antigen-independent responses’ was confirmed, and this concept has permeated basic and clinical research as well as drug development. When ILC2s were discovered, their function in the acute phase of diseases garnered attention because of their rapid and potent type 2 immune response. However, several studies have revealed that the main role of ILC2s is more closely related to the chronicity of diseases, such as allergy and fibrosis, than to the induction of diseases. In this review, we discuss how ILC2 research has affected the concept of ‘*Taishitsu*’, a Japanese term describing the overall nature of an individual as determined by the interaction of genetic and acquired predisposition.

## Introduction

**Fig. 1.**
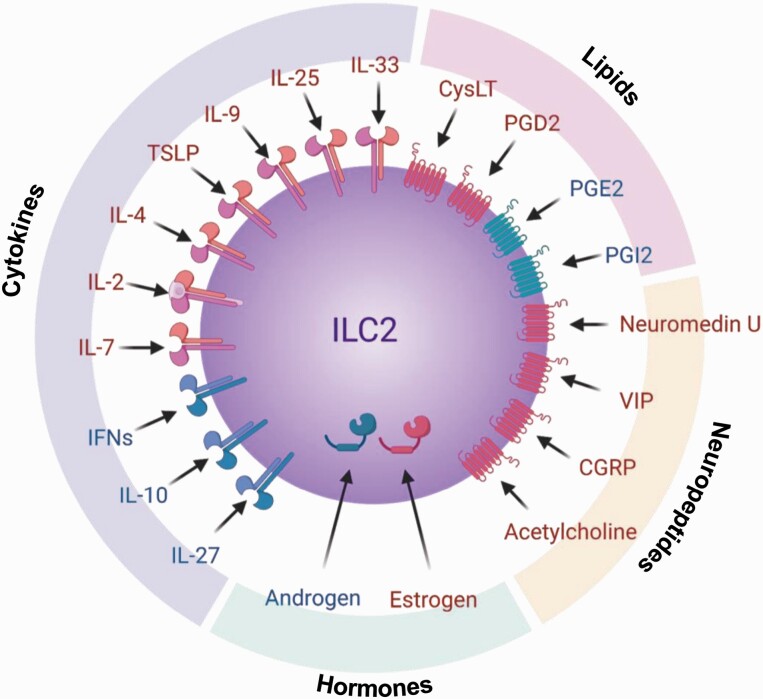
Activators and inhibitors of ILC2s. In addition to the activators IL-25 and IL-33, a variety of cytokines contribute to the activation and inhibition of ILC2s. Not only cytokines, but also lipids, neuropeptides and hormones can regulate ILC2 functions. Red indicates activation and blue indicates inhibition. IFNs, interferons.

Group 2 innate lymphoid cells (ILC2s) were first discovered in adipose tissue (1), but are now known to be present in organs that are exposed to the outer environment, such as the digestive organs, respiratory apparatus and skin, as well as in a variety of tissues, including the liver, muscle and brain (2). However, ILCs are rarely detected in lymphoid tissues under steady-state conditions. ILC2s do not possess antigen-recognition receptors like those on T and B cells and do not elicit antigen-specific responses. However, ILC2s express interleukin (IL)-25 and IL-33 receptors that trigger strong type 2 immune responses. The transcription factor GATA3 is essential for not only cytokine production in ILC2s but also ILC2 development. GATA3 is required for the differentiation of common ILC progenitors (ILCPs) from early ILC progenitors and of ILC2s from ILCPs (3). Progenitor cells migrate to the peripheral tissues in the fetal stage and differentiate and mature in the destination tissues, suggesting that they are tissue-resident cells (4). The function of ILC2s is regulated by not only various cytokines but also physiologically active substances such as lipids, neuropeptides and hormones. Thus, ILC2s constantly monitor the state of the body (Fig. 1).

On the basis of their ability to produce type 2 cytokines, ILC2s were initially thought to have important functions in immune defense against parasitic infections. Shortly thereafter, ILC2s were found to play a major role in the pathogenesis of allergies, and numerous studies focused on the role of ILC2s in allergic pathogenesis (5). However, now it is clear that ILC2s produce IL-5 and IL-13 as well as a wide variety of other cytokines, suggesting that ILC2s are involved in the pathogenesis of not only allergy but also various other immunological diseases. Therefore, ILC2s have garnered attention in clinical medicine (6). In this review, we discuss the long-term nature of ILC2 viability, endogenous factors that activate ILC2s and continuous activation of ILC2s in type 2 immune chronic disease.

## ILC2s display long-term viability

The lifespan of immune cells substantially varies depending on the cell type (7). Many immune cells are programmed to die when they have completed their function; this process is known as activation-induced cell death. Neutrophils last only a few hours in the blood but survive for a longer period when they enter tissues (8). Monocytes survive for 1–2 days but can live for several months by transforming into tissue macrophages (9). Lymphocytes are relatively long-lived immune cells compared with myeloid lineage cells and acquire a memory phenotype that is key for the long-term survival of T and B cells (10, 11). This indicates that the lifespan of cells depends on their surrounding environment, which provides factors that aid in their survival.

IL-7 is essential for the early differentiation of ILC2s, but once they mature, IL-2 can also maintain ILC2s over a long term without activation (1). *In vitro* experiments have shown that ILC2s isolated from adipose tissue survive for 18 months in the presence of IL-2; this effect was not observed in other lymphocytes such as T and B cells. Furthermore, ILC2s did not lose their ability to proliferate or produce type 2 cytokines against IL-33, even after long-term culture. *In vivo*, wild-type ILC2s after transplantation into *γc*^–/–^*Rag2*^–/–^ mice, which lack all lymphocytes, were viable throughout the host’s life (12). Pulse-chase assays, used to measure cell proliferation, revealed that BrdU-incorporated ILC2s survive for more than 4 weeks (13).

Notably, T and B cells survive for long periods by receiving signals from environmental factors, including antigen stimulation that induces switching of metabolic systems and triggers anti-apoptotic mechanisms (14). However, naive ILC2s already have the ability to survive for a long period in the presence of specific cytokines such as IL-2. Fate mapping of IL-2-expressing cells revealed that IL-2 is produced by not only T cells, but also ILC2s and ILC3s (15). Interestingly, ILC2s can return to a steady-state after activation without cell death. In fact, when using a large number of naive ILC2s in experiments, some researchers first expand ILC2s by adding IL-33 and then bring them back to naive cells by switching to IL-2 culture. Even after implementing this protocol, ILC2s did not lose the ability to respond to IL-33 and produced large amounts of type 2 cytokines (16). This property is remarkable and desirable if ILC2s play beneficial roles in the body; however, a different result may occur if ILC2s contribute to diseases.

## ILC2 activation is sustained by a variety of endogenous factors

Although we first reported that ILC2s are activated by IL-25 and IL-33 (1), subsequent studies revealed that cytokines such as IL-4, IL-9 and thymic stromal lymphopoietin (TSLP) are also associated with ILC2 activation (2). Furthermore, lipids such as cysteinyl leukotrienes (CysLT) (17) and prostaglandin D2 (PGD2) (18); neuropeptides such as Neuromedin U (19–21), vasoactive intestinal peptide (VIP) (13) and calcitonin gene-related peptide (CGRP) (22); the neurotransmitter acetylcholine (23, 24); and sex hormones are also involved in activating ILC2s (25) (Fig. 1). These factors are produced in the body, indicating that ILC2s are affected by inflammation, stress and sex differences.

IL-33 is released from epithelial cells that undergo necrosis upon invasion by parasites, fungi and allergens with cysteine protease activity. Cysteine protease is critical for inducing IL-33-dependent inflammation because full-length IL-33 must be cleaved into its activated form by cysteine protease (26–29). Because IL-33 is a nucleoprotein, it was thought that IL-33 acts as an alarmin when released from cells undergoing necrosis (30). However, recent evidence suggests that IL-33 is secreted via endosomes by epithelial cells in chronic obstructive pulmonary disease (31), via Toll-like receptors by vascular endothelial cells (32) and via perforin 2 by dendritic cells (33), suggesting that IL-33 can be produced by cells not undergoing necrosis. These findings should be evaluated in more detail, as epithelial injury from scratching the skin, chronic coughing, or some other endogenous factor may trigger the production of IL-33 and IL-33-mediated responses that indirectly induce necrosis, resulting in a continuous supply of IL-33. This model is understood in terms of sterile inflammation (34). Interestingly, IL-33 is produced in the lungs with the first breath immediately after birth and ILC2s accumulate in an IL-33-dependent manner (35).

Thus, the activation of ILC2s can be sustained by a variety of endogenous factors; even if the intermittent supply of IL-33 is stopped, the expanded ILC2s do not die, as they show longevity and the subsequent supply of IL-33 causes even greater type 2 inflammation.

## Continuous activation of ILC2s triggers type 2 immune chronic disease

Individuals differ in their susceptibility to infections and diseases as well as the degree of severity, even if they have similar diets and lifestyles. This was exemplified by the recent coronavirus disease 2019 (COVID-19) pandemic, in which the age (36) and health status of the host, such as hypertension and obesity (https://www.statista.com/statistics/1111428/covid-hospitalization-underlying-conditions-us/), affected the magnitude and severity of the disease.

‘*Taishitsu*’ is a Japanese term used to describe an individual’s overall susceptibility or resilience to diseases, e.g., having a constitutional tendency to catch a cold or be prone to allergies is affected by the interaction between genetic predisposition and environmental factors. The use of ‘*Taishitsu*’ has been regarded as unscientific because it only expresses a tendency. However, many patients suffer from the ‘allergic march,’ involving stepwise events of different allergic diseases, including atopic dermatitis, food allergy, allergic rhinitis and asthma (37). Some people are repeatedly infected with cold viruses, whereas others rarely are, although they live in the same environment. When siblings eat the same food and perform the same amount of exercise, one sibling may gain weight, whereas the others do not. Although the scientific basis for ‘*Taishitsu’* has not been clearly supported, the high viability of ILC2s and their activation by biological factors may help clarify the mechanisms associated with ‘*Taishitsu*’.

An interesting aspect of allergic susceptibility is that it is not necessarily linked with antigen specificity. Patients with high allergic sensitivity develop different allergies many times in their lifetime and such an allergic march is characterized by the stepwise changes in reactions to antigens. For example, infants with a food allergy to eggs develop rhinitis to pollen at school-going age and then develop asthma to mites in adulthood (38). These facts indicate that the formation of ‘*Taishitsu’* cannot be explained only by T cells with antigen specificity. In contrast, ILC2s are activated by endogenous factors in a non-antigen-specific manner, and they have the potential to form the basis of the next allergy because of their long-term viability and survival after the end of inflammation.

In fact, a greater number of respiratory syncytial virus infections during childhood leads to a greater risk of developing asthma in the future (39); ILC2s play an important role in respiratory syncytial virus-induced asthma exacerbations (40). In addition, ILC2s have a memory-like function; once stimulated by IL-33, ILC2s become more responsive to a second IL-33 stimulation (41). Therefore, the mechanism underlying allergic susceptibility involves the proliferation and activation of ILC2s, which are enhanced in several type 2 immune diseases. Unlike that of T cells, the ability of ILC2s to continuously and homeostatically produce cytokines is an important part of this process (42). Even if each type 2 immune disease is caused by immune cells other than ILC2s and if endogenous factors activate ILC2s, ILC2s can contribute to the development of an allergic state or allergic susceptibility.

## Conclusions

**Fig. 2.**
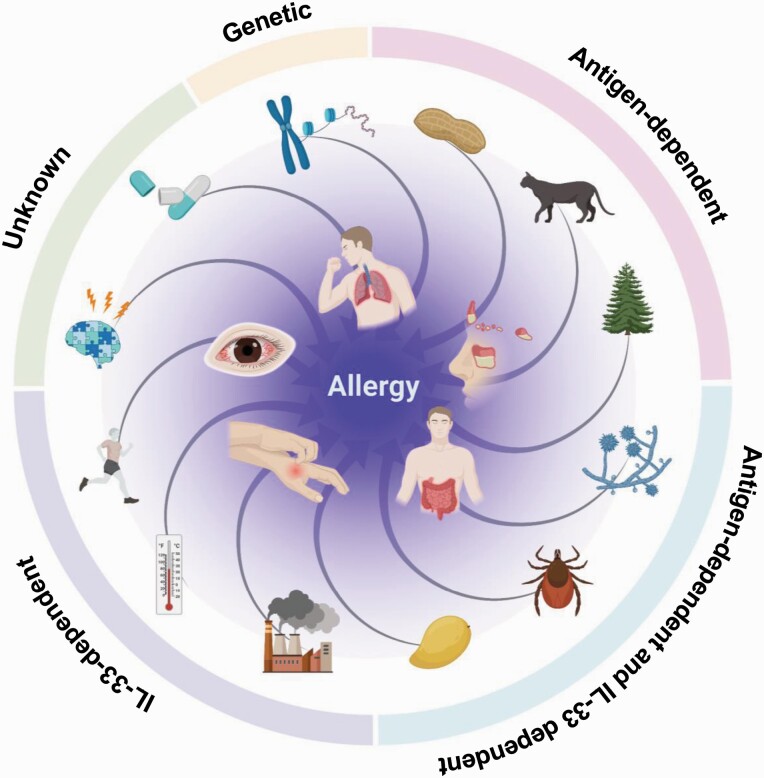
Discovery of ILC2s created a new concept for allergic pathogenesis. With the discovery of the antigen-independent allergic reactions of ILC2s, the pathogenesis of allergy, which was previously explained by adaptive immune T cells, is now thought to be driven by antigen-dependent T cells, antigen-independent IL-33-stimulated ILC2s and a combination of the two cell systems.

A decade ago, ILC2s were reported as cells that rapidly and potently produce type 2 cytokines; since then, studies on ILC2s have changed the concept of allergy, profoundly affecting allergy research. Until the discovery of ILC2s, allergies were considered an antigen-dependent reaction, with the genetic background of an individual influencing the severity of the reaction. This concept is correct for allergies in which the antigens can be identified, such as hay fever and pet allergies. However, in clinical practice, there are many patients for whom the antigen is not identified, and ILC2s may be involved in the condition of these patients (43). For example, fungi, mites and some fruits have antigenic activity; they also induce IL-33-dependent allergies through cysteine protease activity. Furthermore, recent reports showed that air pollutants, cold exposure and exercise also induce IL-33 production, suggesting the involvement of ILC2s in allergies caused by these non-antigenic factors (Fig. 2).

Several studies conducted worldwide have shown that the role of ILC2s spans both acute and chronic phases of a disease. Even in the absence of antigens, ILC2s continue to be activated because of their long-term viability and homeostatic cytokine production, leading to persistent irreversible inflammation events such as allergy and fibrosis. If ILC2s are involved in ‘*Taishitsu’* of type 2 immune response, ILC1s and natural killer cells may be involved in patient susceptibility to viral and bacterial infections (44).

Recently, the term ‘trained immunity’ has attracted attention in the immunological community (45). This concept stems from the perspective that innate immunity can have memory-like mechanisms, which contrasts with the memory of acquired immunity, as invertebrates that do not possess acquired immune cells show stronger responses to re-infection than to initial pathogen infections or transplant rejection (46). Innate immune cells, such as natural killer cells and macrophages, have memory-like ability (47); this type of trained immunity in ILCs must be further examined (48, 49). Although ILC2s cannot ‘remember’ antigens, they may acquire the ability to rapidly re-activate through epigenetic changes, which may also play a major role in the development of allergic susceptibility.

The next decade of ILC2 research will be devoted to studying how the ILC2 activation loop is formed and how it can be broken, which will help establish new therapies that block chronic disease states by targeting the immune activation cycle.
